# Turning over New Leaves

**Published:** 1890-03-15

**Authors:** 


### Turning Over New Leaves.

"James Vraille," *

By Jeffery C. Jeffery, is an incomprehensible mixture of good and bad. So much of it is good, and even excellent, that the inferiority of the rest is all the more extraordinary. The hero is a really fine character, consistently well drawn, and his son, little Jim, is one of the jolliest babies it has been our pleasure to become acquainted with in fiction. Mrs. Foresight, and one or two other minor personages, are human and possible, but with these exceptions Mr. Jeffery has relied upon types, and copied them badly. The first part of the book is weak and amateurish; the names chosen are often silly, and the effect tedious. "Bite, Flight, and Co.," "Mr. Skrim," "Harold Scatter," "Cabstand Square," are examples of this unfortunate punning heraldry. Lucy Flight seems to have been introduced into the story for a specific purpose, which may be worth pointing out. She spoilt her husband's life, and she spoils the book. If the former is good fiction, the latter is certainly bad art; but it is obvious that in wholesome novels a man must have a wife before he can have a son, and little Jim had to be accounted for. Hence the appearance of Mrs. Vraille, who might much better have been "indicated." She has little influence in moulding her husband's character, and the account of her final disappearance with Herbert Rook is commonplace and nauseating. Having once disposed of her, and brought the hero home to England, Mr. Jeffery apparently felt relieved, and began to do good work. In the second volume he shows a command of language, a knowledge of childhood, and an insight into, and power over, human emotion that are all full of promise. Some passages are dramatic in the best sense of the word, and the pathos is genuine without a suspicion of hysteria. The real excellence of the latter half of the story may doubtless counteract the many glaring faults and weaknesses of the earlier part, but it makes them unintelligible. Mr. Jeffery is distinctly to be congratulated, but at the same time it is not impertinent to suggest that careful and critical revision of the book before publication would have made its complete success far more probable.

* "James Vraille: the Story of a Life." 2 vols. By Jeffery C. Jeffery. (W.H. Allen and Co. 1890.)

